# Identifying barriers and facilitators to adopting healthier dietary choices in clinical care: a cross-sectional observational study

**DOI:** 10.3389/fnut.2023.1178134

**Published:** 2023-12-22

**Authors:** Daan L. de Frel, Hope Wicks, Zsuzsa Bakk, Nicole van Keulen, Douwe E. Atsma, Veronica R. Janssen

**Affiliations:** ^1^Department of Cardiology, Leiden University Medical Centre, Leiden, Netherlands; ^2^Section of Methodology and Statistics, Department of Psychology, Leiden University, Leiden, Netherlands; ^3^National eHealth Living Lab, Leiden, Netherlands; ^4^Department of Design, Organization and Strategy, Faculty of Industrial Design Engineering, Delft University of Technology, Delft, Netherlands

**Keywords:** nudge, healthy diet, cardiovascular disease, teachable moment, choice behavior

## Abstract

**Background and aims:**

Adopting healthier diets can drastically improve societal health. Our environment plays a crucial role in daily dietary choices and hospitals in particular can stimulate patients to adopt healthier eating habits. Unfortunately, no robust clinically applicable cuing tools exist to help guide in-hospital dietary interventions. The purpose of this study was to identify patient-related barriers and facilitators to adopting healthier dietary choices.

**Methods and results:**

This cross-sectional observational study was conducted on the cardiology ward of a university medical center between June 2020 and January 2021. Of the 594 patients asked and the 312 completed surveys on healthy eating intentions, 285 responses were considered for analysis. Notably, the majority of respondents were male (68.8%), with an average hospital stay of 3.3 days. The results indicate that cardiac patients attribute significantly greater influence on their dietary behavior to doctors compared to other caregivers, including dieticians (*X*^2^ = 37.09, *df* = 9, *p* < 0.001). Also, younger patients (below 70 years of age) were more inclined to plan changing dietary behavior than older patients. Most mentioned facilitators for adopting a healthier diet were more information/counseling, help in preparing food, support from family and friends, and more emphasis from a doctor.

**Conclusion:**

The study highlights the importance of involving doctors in formulating dietary policies and patient-directed interventions within hospital settings. It also sheds light on the barriers and facilitators for promoting healthier dietary behaviors among patients during their hospitalization.

## Introduction

Cardiovascular disease (CVD) accounts for 24.7% of all deaths in the Netherlands (1). Dutch healthcare expenditures on CVD progressed to over 10 billion euros in 2017, amounting to 11.7% of total healthcare expenditures (2). The elimination of unhealthy risk behaviors such as unhealthy diet, smoking, or physical inactivity may prevent at least 80% of CVD (3). Even changing to a healthy diet alone could reduce the incidence of CVD by as much as 30% (4).

Recognizing the pivotal role of diet in CVD prevention and management, adherence to dietary guidelines has been emphasized as a cornerstone of treatment and prevention strategies for cardiac patients (5–7). However, motivating individuals to adopt healthier behaviors is a complex challenge, and national campaigns have struggled to achieve significant impact, resulting in low adherence rates to dietary guidelines in the Netherlands, estimated at just 20–30% (8, 9).

A crucial factor contributing to this low adherence is the environment in which individuals make their dietary choices (10, 11). The increasing overabundance of cheap, highly processed, convenient, energy-dense, and nutrient-poor foods and drinks contributes to adverse dietary choices (12). These adverse dietary choices can lead to obesity early in life, and CVD, obesity and many other chronic diseases later in life (13, 14). For this reason, our environment has been called “obesogenic” (15). Obesity leads to CVD and CVD mortality, even independently of other CVD risk factors (16). Thus the dietary environment influences CVD incidence and mortality in many ways. Fortunately, our dietary environment can also be part of the solution (17). Many public places such as schools, supermarkets and workplaces can contribute to an environment that nudges toward healthier diets. However, stimulating lifestyle behavior change is especially relevant in healthcare.

It is known that hospitals can influence the health of patients, visitors and employees (18, 19). For example, research has shown that fast food can be perceived as healthier when available within a hospital (20). Therefore, it is seen as the responsibility of the health service to serve as a role model for healthy eating behaviors (18). Fortunately, hospitals collectively gear toward promoting healthier dietary choices. Multiple examples of healthy diet promoting interventions are reported in the literature. A first example are so called nudges, which are environmental cues that are used to help people make healthier dietary choices (21–23). An effective example of using nudging in a hospital setting is a traffic light labeling system that influences healthy food choices in hospital cafeteria (24). Other examples include healthy meal-deliveries after discharge, 100% plant-based menus in hospitals, improvements in food presentation, preparation and purchasing, outpatient education and app-based coaching (25–29). In-hospital interventions are especially important as recent studies show that patients are more susceptible to health-related advice and more prepared to change health-related behavior during admission to the hospital (30, 31). Thus, a hospital stay can and should serve as a “teachable moment.” However, at present it is unclear how to best capitalize on this momentum for change and “what works best for whom.”

Dietary interventions can aim at multiple aspects of behavior to instigate a change. A psychological theory often used to dissect these aspects of behavior is the Theory of Planned Behavior (TPB) (32). According to this theory, intention is the most influential aspect of behavior change and intention is influenced by attitude, subjective norm and self-efficacy. In a previous study we showed the development and tested the internal reliability of a novel questionnaire based on the TPB (33). This questionnaire can be used to explore the normative referents, attitudes and intentions of hospitalized cardiovascular patients. Exploring potential handholds could give direction to future dietary interventions in multiple ways. It could provide information regarding what role should be used to provide the intervention (normative referent). Also the patients most motivated or unmotivated for behavior change (attitude, intention) could be identified and targeted specifically. Lastly, it could further clarify what the intervention should aim for (attitude, subjective norm, and perceived behavioral control).

In light of these considerations, the present study aims to explore potential barriers and facilitators for encouraging patients to embrace healthier dietary choices. Specifically, our research seeks to determine which healthcare providers should deliver dietary advice, identify patient groups most receptive to adopting a healthy diet based on their reason for admission, gender, and age, and map the attitudes, subjective norms, and perceived barriers and motivators of CVD patients. This information will be invaluable in tailoring effective dietary interventions and ultimately mitigating the burden of cardiovascular disease.

## Methods

### Design and study population

This cross-sectional observational study was conducted at the cardiology ward of the Leiden University Medical Center in the Netherlands. All patients admitted to the cardiology ward between July 2020 and January 2021 were invited to participate in the study. Researchers visited the cardiology ward daily to recruit newly admitted patients during their stay. Patients were asked to fill out a one-time, anonymous questionnaire if they were 18 years of age of older and had eaten at least one meal at the hospital ward. Exclusion criteria were absence of email address, insufficient meal consumption (no evening meals consumed), previous participation in this study, inability to provide consent and language barrier. Participants were asked to fill out the questionnaire after their last evening meal in the hospital, this could be done in the hospital or at home. The majority did so in the hospital. Participation to the online survey was on voluntary basis and informed consent was obtained at the beginning of the survey. Castor EDC (Castor, Amsterdam) was used to send and manage the questionnaires (34). All protocols and the process of obtaining informed consent were approved by the Medical Ethics Committee Leiden-Den Haag-Delft.

### Questionnaire

The 20-item long Dutch Dietary Intention Evaluation Tool (DIETI) was used to assess multiple facets of healthy eating. This questionnaire has been specifically developed to assess healthy eating intentions of hospitalized patients and has been found te be reliable (33). The DIETI is based on the TPB and consists of the following subscales; intention (4 items), attitude (5 items), self-efficacy (3 items), subjective norm (3 items) and normative referent (5 items). For the intention, attitude, self-efficacy and subjective norm subscales, 7-point Likert scales were used with higher scores representing stronger intentions, more positive attitudes, higher self-efficacy and higher subjective norms. A scale of 1–10 was used for the normative referent subscale where a higher response endorsed a higher influence. Demographic data included gender, age, reason for admittance, history of cardiac ischemia, healthiness of current diet and number of meals consumed, and were obtained from a self-report questionnaire. The influence of different healthcare professionals were measured using a 10-point scale. The healthiness of current diet was measured using a single question on a 10-point scale where 10 means healthiest and 1 means least healthy. The English and Dutch versions of the questionnaire can be found in Supplementary Table 1 respectively.

### Data cleaning

Data cleaning was performed to identify and correct lacks or excesses of data, outliers and logical inconsistencies. Data entry validation, statistical outlier detection, flatliner detection and fixed algorithms for logical inconsistencies were used. Examples of these algorithms are; age >100 years or a difference > 3 between the number of breakfast- lunch- or evening meals.

### Statistical analysis

Descriptive statistics were used for the demographics. Patients were stratified according to age (based on distribution of age in sample), gender and reason for admission, namely arrythmia, angina/myocardial infarction, heart failure or other. The influence of doctors on dietary behavior of participants was compared to the influence of the other identities and institutions using a chi-square test for trend. The Mann–Whitney–Wilcoxon *U*-test was used to investigate the influence of gender on the various subscales. Moreover, the Kruskal–Wallis test was used to assess the differences in the behavior change subscales between the age groups and reasons of admission followed by the *post-hoc* Dunn multiple comparisons test. Statistical significance was set at p < 0.05 for the chi-square test, the Mann-Whitney-Wilcoxon U test as the Kruskal–Wallis test. All statistical analyses were performed using SPSS Statistics version 25.0 (35). Figures were made using R statistical software and Graphpad Prism version 8.0.0 for Windows (36, 37).

## Results

A total of 505 (out of 594 assessed) patients were deemed eligible and 494 (98%) agreed to participate. Of all participants, 312 (63%) completed the survey, 136 (27.5%) were non-responders and 48 (9.7%) provided incomplete surveys. After data cleaning, 285 responses were used for further analysis (Table 1). The participants had a mean age of 63.1 (*SD* = 12.8) years, were predominantly male (68.8%), had an average hospital stay of 3.3 days (*SD* = 3.8) and the majority was admitted for cardiac arrythmia (42.1%). The mean self-rated healthiness of diet was 7.32 (*SD* = 1.2) on a scale of 1 to 10.

**Table 1:** Characteristics of the study participants (*n* = 285).

**Characteristic**	**Value**
*Total*	285
Age (years), mean (SD)	63 (12.81)
**Age, n (%)**	
< 50	38 (13.3)
50–69	141 (49.5)
70–89	106 (37.2)
**Gender, n (%)**	
Female	89 (31.2)
Male	196 (68.8)
**Reason of admission, n (%)**	
Arrhythmia	120 (42.1)
AP/MI	72 (25.3)
Heart failure	17 (6.0)
Other	76 (26.7)
Admission duration (days), mean (SD)	3.29 (3.79)
Special diet, n (%)	30 (10.5)
Myocardial infarction in medical history, n (%)	77 (27.0)
Diet health score, mean (SD)	7.34 (1.2)

n, number of participants; SD, standard deviation; AP, angina pectoris; MI, myocardial infarction.

### Normative referent

**Figure 1:**
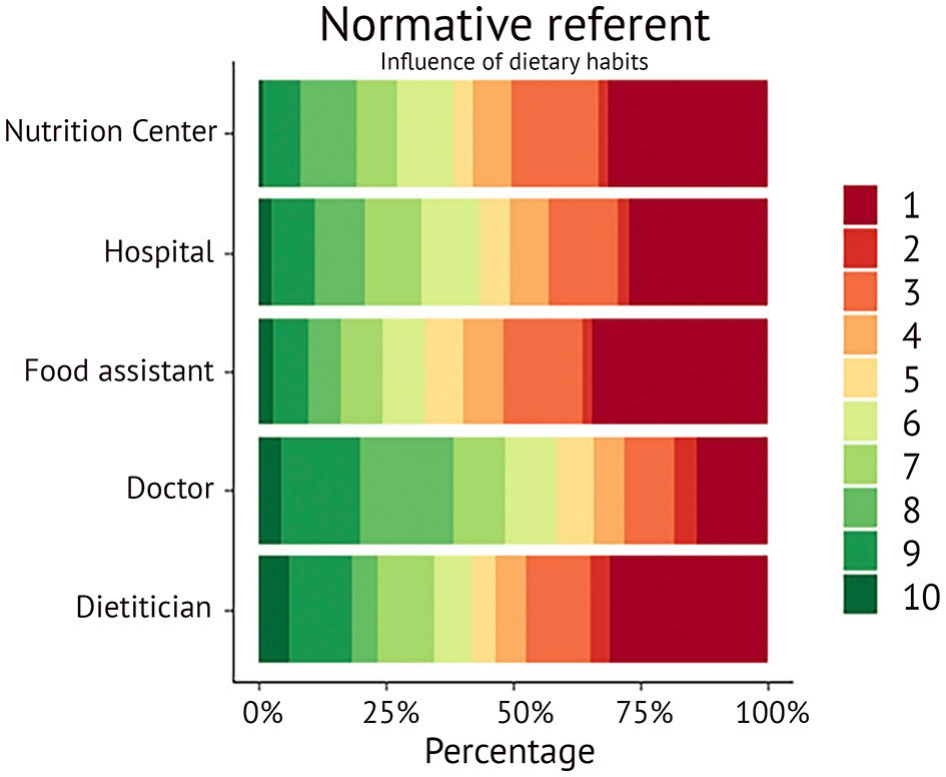
Visual representation of responses of influence on dietary habits by different providers and institutions. The gradient changes from forest green (highest influence of 10) to dark red (lowest influence of 1).

In the DIETI subscale of the normative referent participants were asked to evaluate the influence on dietary habits for various identities and institutions, including the Nutrition Center, hospital dietary policies, food assistant, doctor and dietician. The responses were recorded on a 10-poitn scale and visualized in a color-coded graph (Figure 1). In this graph, green marks indicate higher scores and red marks indicate lower scores. The figure clearly shows that doctors receive the highest number of sufficient marks (≥6, in green). A chi-square test for trend confirms that doctors have a significantly higher influence on dietary behavior than the others combined (*X*^2^ = 37.09, *df* = 9, *p* < 0.001) and even than the dietician separately (*X*^2^ = 24.6, *df* = 9, *p* = 0.003).

### Attitude and intention based on age and gender

The mean scores for the dietary subscales on a scale of one to seven were; intention *M* = 5.23 (*SD* = 1.07), attitude *M* = 5.76 (*SD* = 0.70), subjective norm *M* = 5.06 (*SD* = 0.85), self-efficacy *M* = 5.76 (*SD* = 5.75).

**Figure 2:**
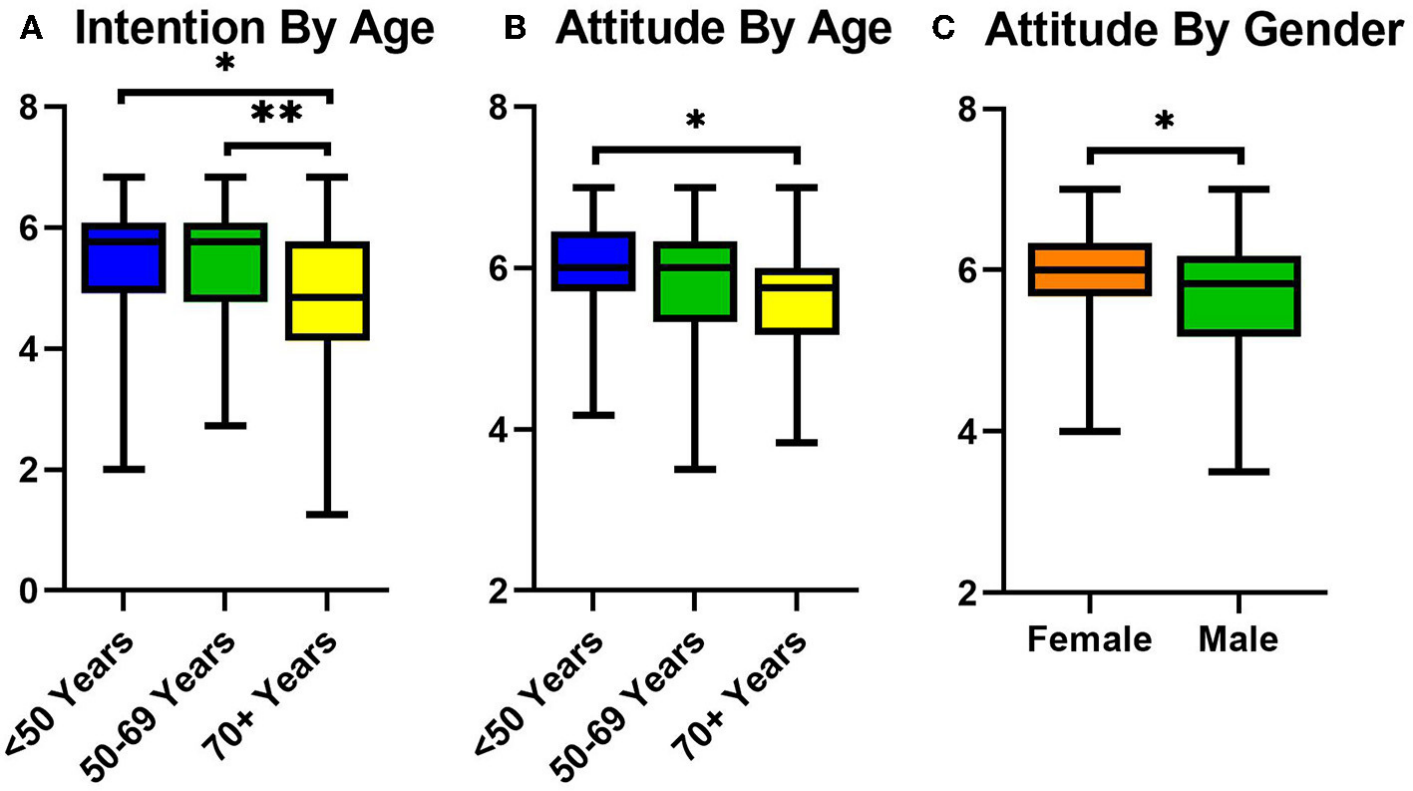
**(A)** Box and whiskers plot showing mean intention for healthy eating across the three age groups; <50 in blue (left), 50–69 in green (middle) and 70+ in yellow (right). **(B)** Box and whiskers plot showing mean attitude for healthy eating across the three age groups; <50 in blue (left), 50–69 in green (middle) and 70+ in yellow (right). **(C)** Box and whiskers plot showing mean attitude for healthy eating classified by gender; female in orange (left) and male in green (right). *p < 0.01, **p < 0.0001.

Based on the distribution of age, patients were divided into three groups, < 50 (*n* = 39), 50–69 (*n* = 138) and 70–89 (*n* = 107) years. Regarding the intention to eat a healthier diet, the youngest group (*M* = 5.46, *SD* = 1.06) had a significantly higher intention than the oldest group (*M* = 4.86, *SD* = 1.07), *U* = 1387.00, *p* = 0.002 (Figure 2). This was also seen when comparing the middle age group (*M* = 5.45, *SD* = 0.95) to the oldest age group (*M* = 4.86, *SD* = 1.07), *U* = 5087.00, *p* = 0.000. A similar difference was seen in the attitude regarding a healthy diet as the youngest age group had a significantly more positive attitude than the oldest age group (*M* = 5.96, *SD* = 0.65 vs. *M* = 5.63, *SD* = 0.69, *U* = 1402.50, *p* = 0.003). Notably, females had a significantly more positive attitude regarding a healthy diet than their male counterparts (*M* = 5.95, *SD* = 0.60 vs. *M* = 5.67, *SD* = 0.72, U = 6679.50, *p* = 0.002). All significant results remained significant after correction of the *p*-value for multiple testing.

### Barriers and facilitators of healthy dietary choices according to patients

**Figure 3:**
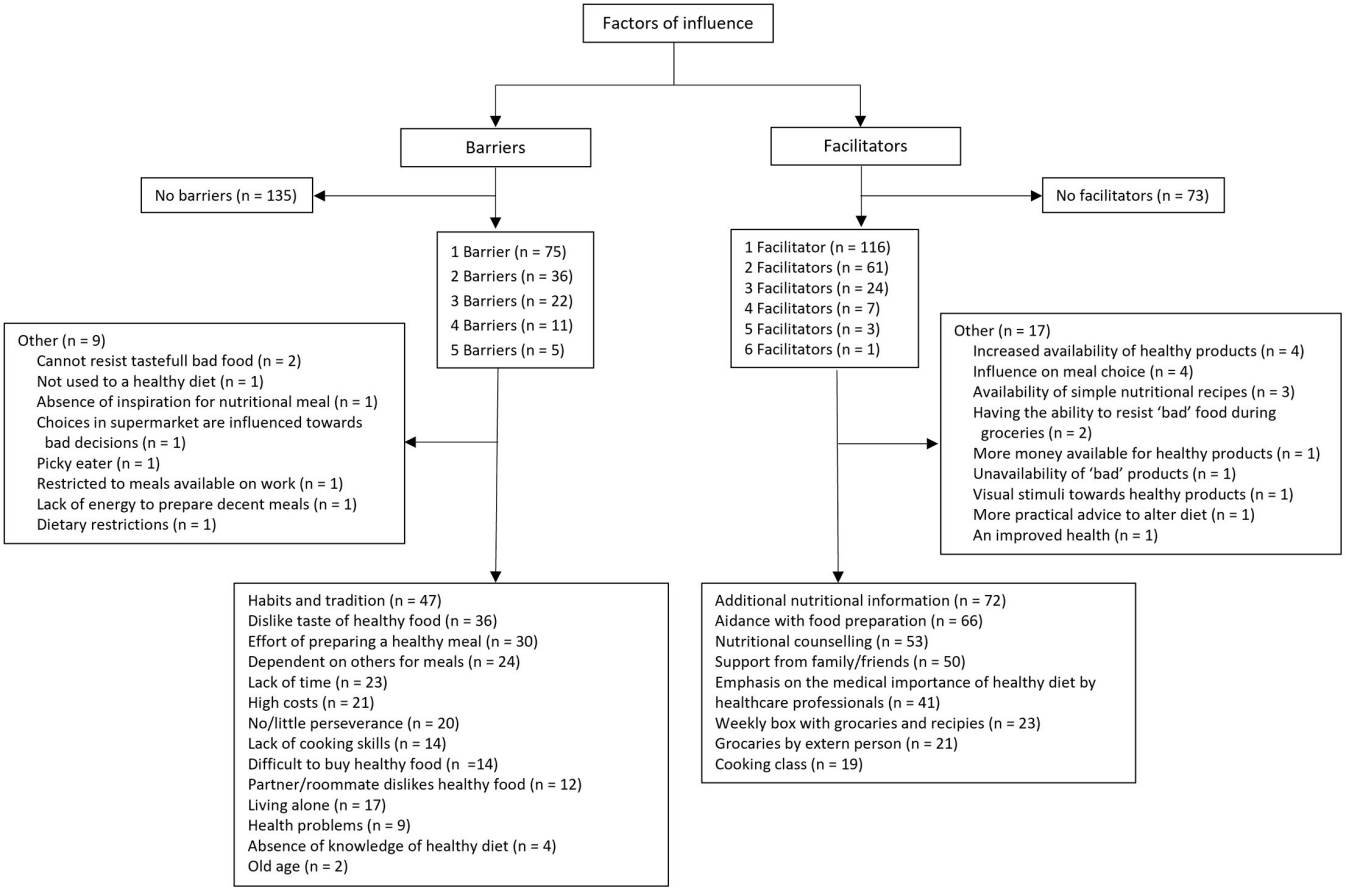
Overview of responses regarding factors of influence. *N* = the number of times a certain response was given.

To gain further insights into attitude, intention, subjective norm, and self-efficacy, participants were questioned about the barriers and facilitators to healthy dietary choices. A total of 150 out of 285 participants reported at least one limiting factor (Figure 3). The most frequently mentioned hindrances to healthy eating included habits and tradition (*n* = 47, 31.3%), a dislike for the taste of healthy food (*n* = 36, 24.0%), the effort required to prepare a healthy meal (*n* = 30, 20.0%), dependency on others for meals (*n* = 24, 16.0%), time constraints (*n* = 23, 15.3%), high costs (*n* = 21, 14.0%), and a lack of perseverance to consistently prepare healthy meals (*n* = 20, 13.3%). A total of 212 (out of 285) participants reported at least one facilitating factor. The most frequently mentioned facilitating factors for altering dietary patterns were additional nutritional information (*n* = 72, 34.0%), a helping hand with food preparation (*n* = 66, 31.1%), nutritional counseling (*n* = 53, 25%), support from family and friends (*n* = 50, 23.6%) and emphasis on the importance of a healthy diet by healthcare professionals (*n* = 41, 19.3%). Other facilitating factors mentioned were weekly boxes with groceries and recipes (*n* = 23, 10.8%), groceries begin brought by someone (*n* = 21, 9.9%) and cooking classes (*n* = 19, 9.0%).

## Discussion

The primary objective of this study was to identify effective strategies for future interventions aimed at promoting healthier dietary choices within clinical care. The key findings of our study are: (1) doctors are the most influential on dietary choices of patients, exceeding all other identities and institutions, and even than dieticians separately, (2) patients rate themselves quite highly on a healthy eating scale, and (3) younger and female patients are more inclined to eat healthy. Below we interpret these results to formulate practical recommendations to guide future interventions aiming to stimulate healthier dietary choices in clinical care.

Firstly, Our study unequivocally highlights the pivotal role of doctors in influencing patients' dietary choices, surpassing other healthcare providers and even dieticians. This is partly in line with a Dutch study conducted in 2015 that asked 1,063 patients to rate the credibility of doctors and dieticians regarding dietary advice. Both roles had very high reliability scores, 95 and 93% respectively (38). However, in our study, doctors were clearly found to be more credible than dieticians when it came to dietary advice. The credibility of a source is important as it can influence the impact of messages on one's health behavior (39). We know that a brief advice from doctors can increase the chance of successful lifestyle alterations in patients in the long term (40). Furthermore, our study resonates with a systematic review of behavior change techniques in cardiac rehabilitation, revealing that interventions emphasizing source credibility correlate with significant improvements in cardiovascular disease risk factors, such as systolic blood pressure and physical performance (41). These results further emphasize that involving a doctor in dietary interventions for in-hospital cardiac patients can increase the effect of the intervention.

Secondly, patients in our study consistently provided high self-ratings regarding their current healthy eating habits, intentions to eat healthier, attitudes toward a healthy diet, and self-efficacy. Previous literature showed that perceived diet quality is overrated when compared to actual diet (42–44), which could mean the self-ratings may be structurally higher than the actual value. While these self-assessments may be influenced by social desirability bias, they remain important indicators for assessing cardiometabolic disease risk. Notably, higher self-rated diet quality has been found to correlate with superior scores on the Healthy Eating Index-2015 and reduced cardiometabolic disease risk factors (e.g., BMI, waist circumference, insulin, cholesterol) (45). So, even though current healthy eating, intention, attitude and self-efficacy seem rather high and might not represent real diet quality, they can be relevant parameters for cardiometabolic disease risk.

Thirdly, our research underscores age and gender as influential factors in motivating dietary behavior change. We found that the intention to eat healthier and the attitude toward a healthy diet were higher for the two younger cardiac patient groups (< 50 and 50–69 years old) compared to the relatively older cardiac patients (age < 70 years). This would suggest that younger cardiac patients are more inclined to alter dietary behavior. These results are in line with previous research that showed younger people in general to be more likely to change behavior compared to older counterparts (46). That young cardiac patients are more inclined to alter behavior emphasizes the general idea that is it essential to start implementing lifestyle medicine as early as possible. Similarly, we found that female patients in our study have a more positive attitude toward healthy a diet. Previous studies also suggested that females in the general population have stronger beliefs in healthy eating (47, 48). Given the predominantly male demographic among cardiac patients, this finding highlights the importance of acknowledging that, generally, male patients may be less motivated to adopt healthier eating habits.

Finally, we explored what dietary interventions should form the target (attitude, subjective norm, perceived behavioral control). When asking patients about limiting factors for healthy eating, the most reported limitations are a habit of unhealthy diets, the dislike of healthy food, and the effort of preparing healthy meals. This is in line with the general idea that breaking old habits should be a considerable part of promoting healthier eating behavior. Stimulating cardiac patients into preparing some healthy meals could enable them to create new habits, appreciate healthy food and lower the threshold for preparing a healthy meal. A total of 19 patients mentioned that cooking classes could stimulate them to eat healthier. Indeed, research has shown that cooking sessions in cardiac rehabilitation are associated with a reduction in myocardial infarctions (49). Furthermore, a study on 28 patients during the COVID-19 pandemic showed that even culinary coaching via Telemedicine can improve cooking skills and promote self-care (50). This suggests that incorporating cooking sessions into healthcare may be an effective way of helping patients change health behavior. When asked for factors that would help them eat healthier, more information/counseling (*n* = 72)/(*n* = 53), help in preparing food (*n* = 66), support from family and friends (*n* = 50), and more emphasis from a doctor (*n* = 41) are mentioned most. Surprisingly, lack of information was only mentioned four times as a limiting factor. We also know that only providing information does not necessarily change behavior (8). However, the results of this study implicate that patients sometimes miss nutritional information. In terms of supportive factors, over a third of patients responded that further emphasis of a doctor would help them eat healthier. This further highlights the need to involve doctors in interventions about healthier diets for patients.

One limitation of this study is the explorative design of the study. Due to privacy regulations we were unable to compare responders to non-responders. This means that with these results we can only speculate about the aim and shape of dietary interventions in hospitals. Another limitation is the narrow study population of cardiac patients in a single center. However, different patient populations in hospitals may be fairly similar in terms of healthy eating intentions.

The main implication of the results of this study is to include doctors in interventions aimed to improve dietary behavior of cardiac patients. At present, even in high-risk patients with CVD, diabetes or hyperlipidemia, only 1 in 5 receive nutrition counseling from their healthcare professional (51). Reasons for the minimal provision of nutrition counseling are lack of training, time, and reimbursement (52). Time and funding may help, but it is also important to note that doctors are not adequately educated in the basics of healthy diets or their promotion (53, 54). Teaching doctors the basics of nutrition counseling and the principles of motivational interviewing with regard to a healthy diet, might be a means to capitalize on the authority of doctors (55). Other recommendations include aiming interventions at patients as young as possible and being aware that female patients might be more inclined to change their behavior compared to male patients. Furthermore, nutritional information, support from family and friends, and cooking sessions are aspects that can support patients in adopting healthier behavior. Even though providing information does not equal behavior change, it might be helpful to have a simple, solid and trustworthy place of information on healthy diets. An example is the website of the Dutch Heart Foundation (Hartstichting) (56).

The results of this study emphasize the need for involving doctors in dietary policies and interventions in hospitals. Further, this study provides handholds for the future dietary interventions in a clinical setting. Future research could focus on conducting trials to evaluate in-hospital patient-centered dietary interventions.

## Data availability statement

The raw data supporting the conclusions of this article will be made available by the authors, without undue reservation.

## Ethics statement

The studies involving humans were approved by the Medische Ethische Toetsingscommissie Leiden-Den Haag-Delft. The studies were conducted in accordance with the local legislation and institutional requirements. Written informed consent for participation in this study was provided by the participants' legal guardians/next of kin.

## Author contributions

DF cleared the study for the ethics committee, wrote the first version of the manuscript, and set up the study with help from NK, DA, and VJ. DF and HW managed participants inclusion and created the figures. HW performed the analyses. ZB checked the statistical analyses. HW, ZB, NK, DA, and VJ performed substantial revisory work on the manuscript. All authors read and approved the final version of the manuscript.
