# Supplementary material for: Identifying barriers and facilitators to adopting healthier dietary choices in clinical care: a cross-sectional observational study
Source: Front Nutr. 2023 Dec 22;10:1178134. doi: 10.3389/fnut.2023.1178134 (PMC10767758; doi:10.3389/fnut.2023.1178134)
Supplement: Supplementary file 1 [file Table_1.docx]

**Supplementary table 1.** Dietary Intention Evaluation Tool for In-hospital patients (DIETI)**. Behaviour change subscales with corresponding questions and statements.**

| **Item** | **Questions* / statements**** |
| --- | --- |
| **Intention, 4 items** |  |
| I1. Willingness of intention | The next month, I will eat healthy/healthier on a regular basis. |
| I2. Plan of intention | The next month, I am planning to eat healthy/healthier. |
| I3. Strength of intention | How strong is your intention to eat healthy/healthier in the next month? (Very weak – very strong) |
| I4. Amount of intention | Of how many meals, of the ten upcoming meals, do you have the intention to eat healthy/healthier? |
| **Attitude towards healthy diet, 6 items** |  |
| I5. Pleasantness | I find healthy eating   - Unpleasant – pleasant |
| I6. Tastefulness | I find healthy eating   - Untasteful – tasteful |
| I7. Importance | I find healthy eating   - Unimportant – important |
| I8. Usefulness | I find healthy eating   - Useless – useful |
| I9. Fitness | Health eating makes me fitter |
| **Self-efficacy, 3 items** |  |
| I11. Knowledge | I know how to eat healthy/healthier |
| I12. Influence | I have influence on the healthiness of my diet. |
| I13. Strength | Even if it gets hard, I will manage to eat healthy |
| **Subjective norm, 5 items** |  |
| I16. Motivation to comply 1 | I find it important what my direct environment thinks about a healthy diet. |
| I17. Injunctive norm 2 | My doctor/hospital think that I should eat healthy. |
| I18. Motivation to comply 2 | I find it important what doctors/hospitals think about a healthy diet. |
| **Normative referent, 5 items** |  |
| I19. Dietitian | To what extent has a dietitian influence on your dietary habits? |
| I20. Doctor | To what extent has a foodservice employee influence on your dietary habits? |
| I21. Food assistant | To what extent has a hospital employee influence on your dietary habits? |
| I22. Hospital | To what extent has a doctor influence on your dietary habits? |
| I23. Nutrition centre | To what extent has the nutrition centre influence on your dietary habits? |

*Questions were presented with a 7-point Likert scale.
**Statements were presented with a 7-point Likert scale ranging from totally disagree – totally agree.
